# Esophageal Squamous Cell Papilloma: A Report of Three Cases

**DOI:** 10.7759/cureus.25115

**Published:** 2022-05-18

**Authors:** Muhammer Ergenç, Taygun Gülşen, Fadime Bahadır

**Affiliations:** 1 General Surgery, Istanbul Sultanbeyli State Hospital, Istanbul, TUR; 2 Pathology, Istanbul Sultanbeyli State Hospital, Istanbul, TUR

**Keywords:** esophagogastroduodenoscopy, esophagus, papilloma, squamous cell papilloma, esophageal papilloma, esophageal squamous papilloma

## Abstract

Esophageal squamous papilloma (ESP) is a rare benign tumor. ESP is generally detected incidentally during esophagogastroduodenoscopy, which is usually performed to investigate dyspepsia. We present three cases of this rare endoscopic finding. While two of our patients were asymptomatic, one had dysphagia, and the lesions were excised. Endoscopists should be able to make the differential diagnosis of papilloma detected in the esophagus and have knowledge about these lesions as they may carry malignant potential. Excision of papillomas is recommended for definitive diagnosis and treatment.

## Introduction

Esophageal squamous papilloma (ESP) is an uncommon epithelial tumor, and it is caught incidentally during an upper endoscopy in most cases. According to the endoscopic series, the prevalence of ESP is between 0.01% and 0.45% and is more common in middle-aged males. Large lesions may present with dysphagia, but ESP is usually asymptomatic. ESP is mainly detected in the middle to lower esophagus. The characteristic endoscopic figure of ESP is a single small, whitish, wart-like lesion grown from the mucosa. However, histopathological examination is needed for a definitive diagnosis [1-4].

The pathogenesis of ESP has not been completely clarified. Gastroesophageal reflux disease (GERD), esophagitis, mucosal irritation, and human papillomavirus (HPV) infection are identified as risk factors. HPV positivity has been reported between 0% and 87.5% in ESPs. Though ESPs are thought to be benign, some publications also report malignant potential [1-4]. We report clinicopathologic features of three esophageal squamous cell papilloma cases detected in a secondary care hospital endoscopy unit.

## Case presentation

Case 1

A 27-year-old female was admitted to the otolaryngology clinic with a history of globus sensation lasting for six months. The patient had a history of allergic rhinitis and was using nasal steroids, and there was no physical examination finding to explain her symptoms. The patient was referred to general surgery for upper gastrointestinal endoscopy. She had an intermittent feeling of difficulty swallowing and nausea. The patient underwent an esophagogastroduodenoscopy and detected a nonulcerated, polypoid, approximately 4 mm in size mucosal lesion in the middle thoracic esophagus. The lesion was excised by biopsy forceps. Pathology reported squamous esophageal mucosa with papillary proliferation, consistent with benign squamous cell papilloma. After excision, the patient's symptoms resolved.

Case 2

**Figure 1:**
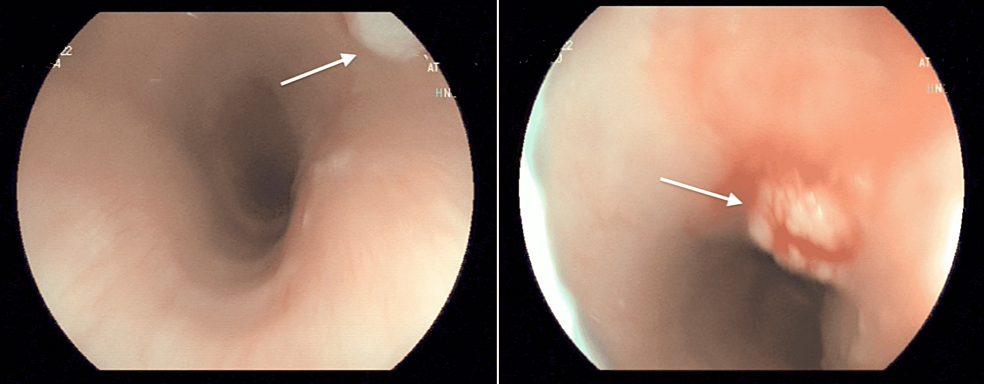
Esophageal squamous cell papilloma of Case 2 Esophagogastroduodenoscopy images showing polypoid lesions with a papillomatous surface (white arrows).

A 36-year-old male patient was admitted to our outpatient clinic with complaints of dyspepsia. He had a history of essential hypertension and irregular antacid use. Esophagogastroduodenoscopy (EGD) showed small papillomas in the upper and middle thoracic esophagus (Figure 1) and antral gastritis concomitant with duodenitis. Esophageal biopsies confirmed findings consistent with squamous papilloma. Gastric biopsy demonstrated complete intestinal metaplasia with low-grade dysplasia and moderate *Helicobacter pylori* positivity. He was treated with antibiotics and an acid-reducing proton pump inhibitor.

Case 3

**Figure 2:**
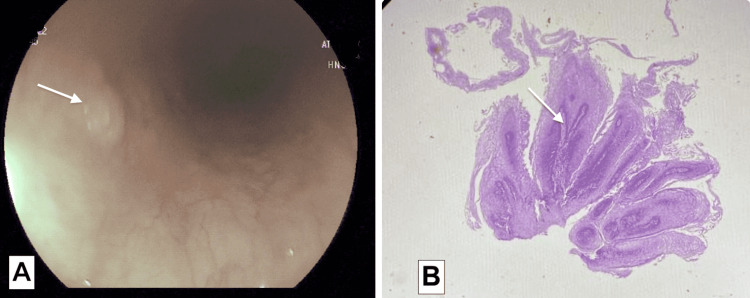
Esophageal squamous cell papilloma of Case 3 (A): Esophagogastroduodenoscopy image showing whitish exophytic growth in the esophageal lumen (white arrow). (B): Histopathological image showing a polypoid lesion with squamous epithelium covering the fibrovascular core (white arrow). Hematoxylin and eosin stain (H&E), 40x magnification.

A 48-year-old female patient with hypothyroidism and a history of peptic ulcer treatment six months ago was evaluated by endoscopy for upper abdominal pain and burning sensation in the stomach. EGD showed a papilloma in the middle thoracic esophagus (Figure 2, Panel A) and multiple ulcers located in the antrum. Esophageal biopsy confirmed findings consistent with squamous papilloma (Figure 2, Panel B).

## Discussion

ESP is a rare benign tumor. ESPs are usually asymptomatic but may present with multiple clinical manifestations such as dysphagia, anemia, and hematemesis. It is generally detected incidentally in EGD, which is usually performed to investigate dyspepsia [5,6]. Although many lesions are detected during EGD, ESP is rarely observed, and it would be beneficial to increase the awareness of endoscopists on this topic [7]. Dysphagia was present in one of our patients, but ESP was found incidentally in two cases. Although international studies report that it is mostly seen in the middle age, the average age is reported as 42 years in the publications from our country. The female to male ratio varies in publications; however, it is more common among females [1,3]. The mean age of our three patients was 37 years, consistent with national studies, and two of our patients were females.

ESPs are usually seen as solitary lesions smaller than 5 mm, but they can be large and multiple [3,8]. Our cases were minor than 5 mm, and one patient had two papillomas. The endoscopic appearance of ESPs can be defined as millimetric solitary lesions originating from the mucosa, small, whitish-light pink with wart-like exophytic projections. These findings are not pathognomonic, and other diseases should be considered in the differential diagnosis, such as verrucous squamous cell carcinoma, granulation tissue, papillary leukoplakia, fibrovascular polyp, and leiomyoma [2,3]. Crossing surface vessels under narrow-band imaging, exophytic growth, and a wart-like shape are characteristic findings for ESPs. The middle esophagus is reported as the most common location; our cases were consistent with these results [4,9].

EPS etiology is not fully determined. Gastroesophageal reflux disease, mucosal damage, esophagitis, HPV infection, alcohol, and tobacco are risk factors for ESP [2,6,8]. The relationship between HPV and ESP is a contentious issue, and the rate of HPV-positive ESP patients is reported in a wide range between 0% and 87.5% in the literature. These differences may be related to geographic variability in the frequency of oral HPV infection and the method of HPV testing [1,2,9]. Unfortunately, we do not know the HPV status of our patients.

Although ESPs are primarily benign tumors, some publications reveal their relationship with squamous cell carcinoma (SCC). The malignant potential is high in patients with multiple lesions (papillomatosis) or a huge lesion. Publications state that it is challenging to identify malignant areas within ESP or papillomatosis in routine endoscopy and biopsies, especially in papillomatosis of the esophagus. There is no consensus on endoscopic surveillance. The standard recommendation for an evaluation of ESP is a biopsy. Several suggested endoscopic treatments for squamous papillomas include forceps biopsy resection, cautery, photodynamic therapies, radiofrequency ablation, and mucosectomy for larger lesions. Esophagectomy can be performed in patients unsuitable for endoscopic follow-up and treatment (cases with extensive dissemination and suspected malignancy) [1,2,6,8].

## Conclusions

ESP is a rare benign tumor. ESPs are usually asymptomatic but may present with dysphagia. Endoscopists should be able to make the differential diagnosis of papilloma detected in the esophagus and have knowledge about these lesions as they may carry malignant potential. Excision of papillomas is recommended for definitive diagnosis and treatment.
